# Rationale for a Multi-Factorial Approach for the Reversal of Cognitive Decline in Alzheimer’s Disease and MCI: A Review

**DOI:** 10.3390/ijms24021659

**Published:** 2023-01-14

**Authors:** Rammohan V. Rao, Kaavya G. Subramaniam, Julie Gregory, Aida L. Bredesen, Christine Coward, Sho Okada, Lance Kelly, Dale E. Bredesen

**Affiliations:** 1 Apollo Health, Burlingame, CA 94011, USA; 2 Department of Psychology, University of California, Davis, CA 95616, USA; 3 Department of Molecular and Medical Pharmacology, University of California, Los Angeles, CA 90024, USA

**Keywords:** Alzheimer’s disease, cognitive decline, AD risk factors, therapeutics, diet, exercise, sleep, brain stimulation, stress, supplements, herbs, neurodegeneration, multi-therapeutic program

## Abstract

Alzheimer’s disease (AD) is a multifactorial, progressive, neurodegenerative disease typically characterized by memory loss, personality changes, and a decline in overall cognitive function. Usually manifesting in individuals over the age of 60, this is the most prevalent type of dementia and remains the fifth leading cause of death among Americans aged 65 and older. While the development of effective treatment and prevention for AD is a major healthcare goal, unfortunately, therapeutic approaches to date have yet to find a treatment plan that produces long-term cognitive improvement. Drugs that may be able to slow down the progression rate of AD are being introduced to the market; however, there has been no previous solution for preventing or reversing the disease-associated cognitive decline. Recent studies have identified several factors that contribute to the progression and severity of the disease: diet, lifestyle, stress, sleep, nutrient deficiencies, mental health, socialization, and toxins. Thus, increasing evidence supports dietary and other lifestyle changes as potentially effective ways to prevent, slow, or reverse AD progression. Studies also have demonstrated that a personalized, multi-therapeutic approach is needed to improve metabolic abnormalities and AD-associated cognitive decline. These studies suggest the effects of abnormalities, such as insulin resistance, chronic inflammation, hypovitaminosis D, hormonal deficiencies, and hyperhomocysteinemia, in the AD process. Therefore a personalized, multi-therapeutic program based on an individual’s genetics and biochemistry may be preferable over a single-drug/mono-therapeutic approach. This article reviews these multi-therapeutic strategies that identify and attenuate all the risk factors specific to each affected individual. This article systematically reviews studies that have incorporated multiple strategies that target numerous factors simultaneously to reverse or treat cognitive decline. We included high-quality clinical trials and observational studies that focused on the cognitive effects of programs comprising lifestyle, physical, and mental activity, as well as nutritional aspects. Articles from PubMed Central, Scopus, and Google Scholar databases were collected, and abstracts were reviewed for relevance to the subject matter. Epidemiological, pathological, toxicological, genetic, and biochemical studies have all concluded that AD represents a complex network insufficiency. The research studies explored in this manuscript confirm the need for a multifactorial approach to target the various risk factors of AD. A single-drug approach may delay the progression of memory loss but, to date, has not prevented or reversed it. Diet, physical activity, sleep, stress, and environment all contribute to the progression of the disease, and, therefore, a multi-factorial optimization of network support and function offers a rational therapeutic strategy. Thus, a multi-therapeutic program that simultaneously targets multiple factors underlying the AD network may be more effective than a mono-therapeutic approach.

## 1. Introduction

Alzheimer’s disease (AD) was officially listed as the sixth-leading cause of death in the United States in 2019 and is the fifth leading cause of death among Americans aged 65 and older [1]. As it has impacted the lives of the elderly around the world, it is now more important than ever to develop an effective treatment not only to slow the progression of this disease but also to create a preventive approach [2,3,4,5,6].

The major genetic risk factor for AD is apolipoprotein E, epsilon 4 allele (ApoE4), and the inheritance of one or two copies of ApoE ε4 increases AD risk approximately 3- or 12-fold, respectively [7]. Based on recent findings, it appears that ApoE4 acts as a transcription factor and binds to the promoters of genes involved in a range of processes linked to AD disease pathogenesis. Interestingly, several of these genes have previously been linked to AD pathogenesis and include genes involved in inflammation, energy metabolism, cardiovascular disease, estrogen regulation, axon guidance, neuronal survival, and cell death [8,9,10,11]. The transcriptional role of ApoE4 needs further investigation since it influences brain health and homeostasis beyond the known β-amyloid or Tau pathways, thus pointing to novel therapeutic strategies for AD [8,12,13,14].

While the development of effective treatment and prevention is a major healthcare goal, unfortunately, therapeutic approaches to AD to date have not led to sustainable improvements. The best results from recent clinical trials have been to delay the progression of cognitive decline rather than improve cognition or halt the decline [15,16]. More than two hundred promising drug candidates have failed clinical trials in the past decade, suggesting that AD and its causes may be complex [15,16]. Some of the potential reasons for failure in effective drug development include the following: (1) AD starts out with a long pre-symptomatic period, but treatment is typically initiated late in the pathophysiological process [17]; (2) it appears that AD is not a single disease as it exhibits several different subtypes [18]; (3) there may be multiple potential contributors to AD, such as inflammation, toxins, infections, trophic withdrawal, insulin resistance, vascular compromise, and trauma [2,18,19,20]; (4) The model of AD on which the drug targets are based (e.g., amyloid-β peptide, tau) is incomplete owing to several potential contributors [15,16].

**Figure 1:**
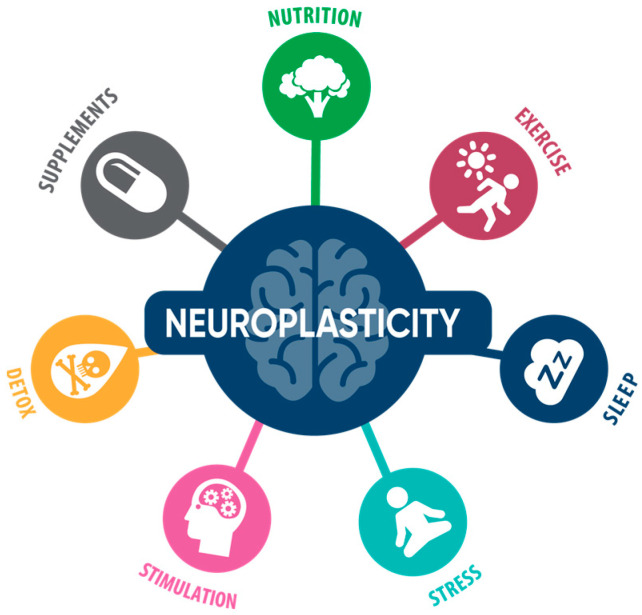
Identifying and addressing potential contributors to cognitive decline with a personalized, multi-therapeutic approach is supportive of cognitive health. Shown in the figure are the various core strategies for the reversal of AD and MCI and for optimizing brain health.

Based on recent evidence from a number of independent groups, it appears that AD is unique to each individual and, in different individuals, has different genetics, epigenetics, biochemistry, subtypes, and, thus, different responses to treatment. Several of the recent clinical trials and observational studies showed superior outcomes when a multitude of these potential contributors was taken into account and addressed simultaneously. Table 1 shows that, given the complex nature of AD pathophysiology, a “perfect” drug may be required to be highly multi-functional. Thus, identifying and addressing all potential contributors to cognitive decline with a personalized, multi-therapeutic approach may be a more effective disease-modifying strategy [5,18,21,22,23,24,25,26,27,28]. Shown in Figure 1 are the various strategies for the reversal of AD and optimization of brain health.

This article systematically reviews clinical trials and observational studies that have incorporated multiple strategies to target numerous factors simultaneously to reverse, prevent, or treat cognitive decline and dementia.

## 2. Multiple Strategies to Optimize Brain Health

### 2.1. Diet & Nutrition

A healthy dietary management strategy including dietary patterns, food, and dietary supplements may be a component of an effective protocol to prevent MCI or AD-associated cognitive impairment. Since gut microbiome and other gastrointestinal (GI) issues, metabolic syndromes, such as diabetes and obesity, gut inflammation, and oxidative stress have long been considered to play major roles in cognitive impairment and AD, not surprisingly, most of the research studies focusing on diet and dietary intervention trials have involved foods or dietary supplements that addressed the above issues [29,30,31,32]. There is sufficient evidence from epidemiological and observational studies and randomized controlled trials (RCTs) that suggest a neuroprotective role of the Mediterranean diet, the Dietary Approaches to Stop Hypertension (DASH) diet, the Mediterranean-DASH Intervention for Neurodegenerative Delay (MIND) diet, and the KetoFLEX 12/3 diet in reducing cognitive decline [2,19,32,33,34,35,36,37,38,39,40].

The Mediterranean, DASH, and MIND diets encompass a multi-nutrient dietary profile that includes fruits, vegetables, nuts, cereals, legumes, olive oil (as the main source of fat), moderate consumption of fish, and a low to moderate intake of dairy products, red meat, and meat products [40,41,42].

The DASH diet also emphasizes foods that are low in sodium and rich in potassium, calcium, and magnesium [43]. Higher adherence to these diets was associated with better cognitive function, lower rates of cognitive decline, and reduced risk of AD [44,45,46,47]. Furthermore, certain food groups included in these various diet options, such as fruits and vegetables, legumes, whole grains, nuts, and olive oil, are by themselves known to improve cognitive functioning [47,48,49,50,51,52]. Specific nutrients like unsaturated fatty acids, antioxidants, and dietary flavonoids have also been associated with better cognitive functioning and a lower risk of cognitive decline in the follow-up period [53,54,55,56].

The Finnish Geriatric Intervention Study to Prevent Cognitive Impairment and Disability (FINGER) program, which was initially developed to improve heart and vascular health, relies on a diet that includes fish, fruits, vegetables, and oils. The intervention also includes physical exercise, cognitive training, social activities, and the management of vascular and metabolic risk factors. This multi-domain lifestyle intervention prevents or slows down cognitive decline. While the participants in the FINGER trial did not exhibit any cognitive issues, they had an increased risk for dementia based on vascular risk factors. While improvement was observed in both the intervention and the placebo groups, the improvement was much greater and better sustained in the intervention group in all cognitive tests administered, namely executive function, information processing, and complex memory tasks. The risk of cognitive decline was 30% higher for the control group compared to the intervention group [4,5,57].

The KetoFLEX 12/3 diet is a part of the ReCODE Program, and unlike other programs (FINGER, DASH, etc.), it is a precision medicine approach that utilizes seven foundational strategies, as well as targeted therapeutics for identified pathogens, toxins, deficiencies, and immune dysfunction, to optimize brain health [2,19]. KetoFLEX 12/3 is a plant-rich ketogenic diet that has proved to be an important component of an effective strategy for the reversal of cognitive decline associated with AD and MCI health [2,19,58]. It is a heavily plant-based, nutrient-dense, whole-food diet that emphasizes local, organic, and seasonal non-starchy vegetables from every color of the rainbow, combined with an adequate amount of protein and generous amounts of healthy fat. KetoFLEX 12/3 also incorporates a long daily fast—a minimum of 12 h, with at least 3 h of fasting before bedtime. This approach utilizes multiple mechanisms to support the brain to prevent and reverse cognitive decline, such as increased energy (via ketosis), insulin sensitivity, reduced inflammation, improved vascular health, and detoxification [2,19,59].

Patients with cognitive impairment and the onset of AD who enrolled in the ReCODE program showed significant improvement in cognitive functioning with outcome measures such as Montreal Cognitive Assessment (MoCA), AQ-21 (a subjective scale completed by the significant other or study partner), and AQ-C change scale (a subjective scale of cognitive improvement or decline completed by the significant other or study partner) [2]. Furthermore, participants in the ReCODE program experienced improved metabolic parameters and cognition, resulting in either arrested cognitive decline or, in most cases, improved cognitive performance [2,19].

### 2.2. Physical Exercise

Physical exercise (PE) has been proven to help prevent and remediate cognitive decline [60,61,62]. Research studies have shown that a consistent practice of physical activity is associated with a lower risk of cardiovascular disease and physically active individuals are less likely to develop dementia [60,63,64]. Research at the cellular and molecular levels coupled with in silico studies suggested that physical exercise impacts brain health by regulating and enhancing genes, proteins, and other neurotrophic factors that directly affect memory, mood, assimilation, and growth [65,66,67,68,69,70]. Specifically for brain health, three types of exercise are recommended:

(1) Aerobic exercise increases heart rate and oxygen uptake, thereby improving cardiovascular health, which in turn benefits the brain. Studies have shown that aerobic exercise improves blood flow to the brain and stimulates the release of brain-derived neurotrophic factor (BDNF), which promotes neuroplasticity, thereby preserving brain volume. Moderate to vigorous aerobic exercise also activates the glymphatic system that promotes the clearance of β-amyloid and other toxins.
(2) Strength training physical exercises improve muscle strength, muscle mass, and endurance, thereby preventing sarcopenia. Furthermore, strength training exercise improves higher-level cognitive processes and memory, stabilizes brain volume, and decreases white matter lesions.
(3) Mind-body exercise combined with body movement improves balance, coordination, gait, and agility; improves neuronal, synaptic, and vascular systems of the brain; promotes the connectivity of brain regions, thereby improving executive function, memory, and emotional status; and curbs neuronal inflammation, all of which improve brain health [26,27,37,61,62,71,72,73,74,75,76,77,78,79,80,81,82,83].

A multifactorial intervention involving diet, PE, and other lifestyle changes may be more effective for ameliorating cognitive decline and may have a sustained beneficial effect that is more pronounced than a single intervention [21,84,85]. While adherence to a Mediterranean-type diet and higher physical activity was associated with reduced risk for dementia, the FINGER trial and the ReCODE program clearly demonstrated that physical exercise in combination with other interventions, including vascular risk management, diet, and cognitive training, was associated with improved cognitive performance. Among the best improvements were those seen in cognitive functions, cognitive impairment, overall health, and mood status [2,4,19,84].

### 2.3. Sleep

The core functions of sleep are to repair, reorganize, maintain brain health, and clear waste [86,87,88]. Sleep facilitates memory consolidation and new learning while also laying down new memories as long-term memories [89,90]. Often sleep disturbances will appear in the preclinical phase of AD. Cognitive decline and an increased risk of mild cognitive impairment and dementia are associated with poor sleep quality [91]. Older adults with disturbed sleep experience a faster decline in cognition than those who sleep well. It is now known that people with AD often have sleep difficulties, and the lack of sleep may, in turn, influence Alzheimer’s-related brain changes that can begin several years before memory loss and other AD symptoms appear. Poor sleep or sleep deprivation triggers β-amyloid build-up in brain regions that include the thalamus and hippocampus, which are vulnerable to damage in the early stages of AD. Study participants with elevated levels of β-amyloid reported mood disturbances after sleep deprivation [92,93,94]. Researchers have suggested that a wide variety of cognitive functions, ranging from attention and memory to language and reasoning, are affected by the lack of adequate sleep [95,96,97].

AD may be accompanied by other co-morbid medical conditions or behavioral disorders which can contribute to sleep issues. Conditions including but not limited to restless legs syndrome and mental health disorders, including anxiety and depression, are all associated with sleep difficulties. Certain medications, such as decongestants, steroids, and some medicine for high blood pressure, asthma, and depression, can also trigger sleep disruptions [86,98,99,100,101,102]. Nearly 15% of AD cases may be attributed to sleep problems that can also be due to the long-term use of benzodiazepines [91,103,104]. Further evaluation of the neurophysiological and cellular mechanisms by which poor sleep contributes to AD may help in identifying new molecular targets for intervention [105,106,107,108].

Melatonin is a sleep-influencing, circadian-rhythm-dependent neuroendocrine hormone that has a protective role in the development of AD because of its anti-inflammatory and antioxidative effects [109,110,111]. Studies have shown that healthy subjects have higher levels of melatonin compared to AD patients [112,113,114]. Melatonin acts as an anti-oxidant by blocking free radical production, reduces Aβ- and NFκB-induced inflammation and, thus, serves as an attractive therapeutic candidate for AD [115,116,117,118,119,120]. Thus, combined with a proper diet, PE, and other lifestyle changes, sleep intervention may be more effective in ameliorating cognitive decline and may have a sustained beneficial effect that is more pronounced than a single intervention [2,19].

### 2.4. Mind and Mental Exercise

Several research studies have now clearly shown the remarkable ability of the brain to reorganize and network in response to various sensory experiences. This neuroplasticity involves adaptive structural and functional changes to the brain [121]. Both healthy and diseased brains have the ability to change their activity in response to intrinsic or extrinsic stimuli by reorganizing their structure, functions, or connections following mental stimulation, brain training, or even traumatic injuries [122,123,124]. AD is characterized by altered hippocampal synaptic efficacy leading to synaptic dysfunction, neuronal degeneration, and cognitive impairment [125,126]. During the pre-AD phase, individuals usually present with mild cognitive impairment [127,128,129]. In the mild to moderate stages of AD, cognitive impairment becomes more profound and widespread, and functional disability becomes increasingly evident—particularly in relation to more complex activities [130,131,132]. In the more advanced stages of AD, most cognitive and functional abilities are profoundly impaired [133].

One of the most important sensory activities is the sense of sound, which has the power to stimulate the brain, which is why hearing loss has a profound impact on brain health. Recent studies suggest that hearing loss causes brain changes that raise the risk for AD. Individuals with moderate to severe hearing loss are up to five times as likely to develop AD-associated dementia, though more research is needed to determine the exact connection between sound, hearing loss, and AD [134,135,136].

AD also features emotional disturbances, including depression, anxiety, irritability, and apathy, that are commonly observed during the mild–moderate stage of AD. Mood disorders in AD patients are also associated with structural changes in the hippocampus, entorhinal cortex, and other regions of the temporal lobe. Researchers believe that mood disorders trigger inflammation and disturb the normal balance of neurotransmission, leading to microglial activation and neurofibrillary tangle formation, which result in neuronal loss, suggesting the need for mental stability and mood stabilizing strategies [137,138,139,140].

Results from several observational studies and randomized clinical trials have indicated that people who engage in cognitively stimulating activities may show improvement in moods, thinking, hearing, problem-solving, reasoning, and memory and have a lower risk of cognitive decline and dementia. Improvement was also seen in activities of daily living (ADLs) [122,141,142,143,144,145].

The ReCODE program clearly demonstrated that cognitive training, together with the other interventions, was associated with improvement in MoCA scores, CNS Vital Signs Neurocognitive Index, and AQ-C. The cognitive improvements were sustained, and no serious adverse events were recorded [2,19].

### 2.5. Stress Management

Research studies have shown that stress is one of the key factors involved in the development of AD [146,147]. In animal models of AD, stress, in large part, activates the hypothalamic-pituitary-adrenal (HPA) axis, which in turn elevates circulating corticosteroid levels [147,148,149,150,151]. The dysregulation of the HPA axis and elevated levels of cortisol are commonly seen in people with AD [148,151,152,153,154]. Stress disrupts the balance between the cortisol receptors (glucocorticoid and mineralocorticoid) that are present in the hippocampal area, leading to atrophy and the degeneration of the hippocampus [155,156,157,158,159]. Stress affects other biological pathways as well, including the brain’s immune system, by producing pro-inflammatory cytokines, thereby promoting inflammation, which underlies AD pathogenesis [160,161,162]. These findings suggest that stress management is critical to maintaining optimal cognitive health.

Stress management techniques for people with AD that are effective in improving the subjective well-being state include breathing exercises, mindfulness techniques, meditation, yoga, tai chi, spiritual practice, socialization, and other activities that focus on the present moment rather than allow distractions of continuous thoughts and mental turbulence [83,163,164,165,166,167,168,169,170,171,172,173].

Other interventions to mitigate lifestyle stressors are dance and music. These interventions have proven to be useful in improving verbal fluency and language ability in patients with AD, specifically those with MCI. There is growing evidence that music and dance reduce stress, increase cognitive acuity, promote a sense of well-being, and improve health span [174,175,176,177]. Dance movements involve a lot of physical activity. Furthermore, dance steps, arm patterns, formations, speed, and various rhythmic movements keep the subjects in a constant mental learning process [178,179,180]. The most challenging aspect of dance training required the subjects to recall the dance steps and routines in a timely manner [181]. While all forms of dance reduce stress, improve cardiovascular health, and stimulate social connectivity, some dance forms that involve split-second changes in steps and complicated moves have an advantage over others when it comes to boosting cognitive acuity [178,179,180,182,183]. Dancing involves continuous learning, which improves the kinesthetic, rational, musical, and emotional aspects of the brain, and ultimately promotes neural connectivity [184,185].

Additionally, music therapy (playing or listening) and art (drawing, painting, and sculpture) improve the quality of life and cognitive and emotional functions. Furthermore, these interventions have also improved stress, mood, well-being, sleep, and the quality of life in adults with subjective cognitive impairment (SCI), MCI, or AD. Significant improvements in anxiety and depression were also observed, and in all these cases, the physical and cognitive benefits were sustained [173,186,187,188,189,190,191,192].

The ReCODE program also reiterates the importance of stress management, which emphasizes regular deep breathing exercises and regular brain training [2,19].

### 2.6. Toxicity and Detoxification

Toxins are increasingly recognized to raise the risk of developing AD [193,194,195,196,197]. Specific toxins that can lead to dementia are called dementogens and include metals, organic chemicals, and biotoxins [198,199,200,201,202]. Research studies show that most people have varying levels of these toxins within their bodies, which can have a deleterious impact on brain structure and function [197,203,204,205].

#### 2.6.1. Metal Toxicity

Mercury, aluminum, arsenic, lead, and cadmium are associated with numerous health issues, even at low levels of exposure. Although manganese, iron, zinc, and copper are essential metals, toxic levels can be harmful. The neurotoxicity of these metals and their roles in AD pathology have been documented in cell and animal models. Human epidemiologic studies have shown a close relationship between elevated levels of these metals and impaired cognitive function and cognitive decline [193,194,206,207].

#### 2.6.2. Chemical Toxicity

In addition to metal toxicity, chemical toxicity that is a risk for developing AD arises from exposure to inorganic and organic hazards, which include pesticides (e.g., organochlorine and organophosphate insecticides), industrial chemicals (e.g., flame retardants), and air pollutants (e.g., particulate matter). Long-term exposure and the bioaccumulation of these environmental chemicals trigger neuroinflammation and neuropathology, paving the way for developing AD [208,209]. Chronic exposure to chemical toxins triggers the reduction in volumes of the hippocampus and total gray matter. Brain imaging studies have also found that the areas of the brain most vulnerable to the toxic effects of chemicals and other environmental toxins are the pre- and post-central gyri, temporal transverse gyrus, and the calcarine regions. While epidemiologic associations between environmental chemical exposure and AD are still limited, the risk of developing AD in older adults due to neurologic impairments caused by environmental toxins is well established [199,210,211,212,213].

Studies in cell and animal models have revealed alterations in neural pathways and metabolism associated with AD. Neuro-imaging studies have reported associations between exposure to toxic chemicals and white matter volume reduction [213,214,215]. Other reported effects include reduced gray matter, larger ventricular volume, and smaller corpus callosum. In addition, studies have also reported associations between a range of chemical pollutants and effects on cognitive function in older people, including the acceleration of cognitive decline and the induction of dementia [216,217,218,219,220,221].

#### 2.6.3. Infections and Biotoxins

Recent studies have provided overwhelming evidence about the possibility of an infectious etiology for AD. The infiltration of the brain by pathogens, including but not limited to B. fragilis, HSV-type 1, *Chlamydia pneumoniae*, and *P. gingivalis*, is most frequently implicated in AD pathogenesis [204,222,223,224,225,226,227]. These pathogens may directly cross a weakened blood–brain barrier and trigger neurological damage by eliciting neuroinflammation. Alternatively, increased gut permeability induced by gut microbiota may promote AD. Inflammatory microorganisms in gut microbiota are associated with peripheral inflammation in subjects with cognitive impairment [222,225,226,228,229,230]. *Chlamydia pneumoniae* can infect the central nervous system via the olfactory and trigeminal nerves resulting in the dysregulation of key pathways involved in AD pathogenesis. Similarly, bacteria can travel from infections in the mouth through the bloodstream to the brain, and this is one mechanism influencing the cascade of events that leads to dementia. Older adults with signs of gum disease and mouth infections were more likely to develop antibodies against the oral bacterium *P. gingivalis*, which could cluster with other bacteria, such as *C. rectus* and *P. melaninogenica*, to further increase the risk of developing AD [205,224,231,232,233].

Similarly, viruses including Herpes simplex 1 (HSV-1) and Varicella zoster virus (VZV) activate the NF-kB-pro-inflammatory signaling system and have been associated with an increased risk of AD. This suggests that AD can be mitigated using appropriate antivirals for treatment or just possibly for prevention [234,235]. Given the pro-inflammatory nature of the type 4 allele of the apolipoprotein E gene (APOE-ε4), this population may especially benefit from an antiviral regimen [223,227,236,237,238,239,240].

Additionally, while mold exposure has historically been connected with asthma and lung disease, mold-exposed people have reported impaired memory and concentration [197,241,242,243]. Mold toxins, including trichothecenes from *Stachybotrys*, aflatoxin from *Aspergillus*, and ochratoxin A from *Aspergillus* and *Penicillium*, are risk factors for the progression of AD because of their neurotoxic effects and ability to impair cognitive functioning [197,230,241,242,243,244]. Researchers are beginning to outline the specific inflammatory pathways by which mold affects the brain, particularly in relation to type 3 (toxic) AD [197]. Mold spores trigger the body to mount an immune response, and people who develop chronic inflammation (including brain inflammation) following mold exposure are most likely to experience cognitive decline [197,230,241,245].

Thus, preventing and treating dementogen exposure and limiting ongoing exposure are paramount to optimizing brain health. Detoxification needs to be an integral part of any personalized, multi-therapeutic program to address overall health optimization and improve cognition [2,19,197].

### 2.7. Supplements & Neuroprotective Herbs

Neuroprotective herbs and supplements have great potential as part of an overall program for preventing and treating cognitive decline associated with MCI and AD. Numerous medicinal plants and their constituents are recommended to enhance cognitive function and alleviate other symptoms of AD, including poor cognition, memory loss, and depression [246,247]. A single herb or a mixture of herbs is normally recommended, depending on the severity of the condition. The rationale is that the bioactive principles present in the medicinal plant act synergistically and modulate the activity of other constituents from the same plant or other plant species [248,249]. Numerous plants and their constituents are reputed in traditional practices of medicine to enhance cognitive function [246,247,250,251]. This approach has been used in Ayurveda, traditional Chinese medicine (TCM), and the Native American system of medicine, where a single herb or a combination of two or more herbs is commonly prescribed [249,252,253,254,255].

Various herbs that inhibit acetylcholinesterase activity, improve cholinergic function, possess anti-inflammatory and antioxidant activities, contain natural COX-2 inhibitors, protect against brain cell degeneration, help in the reduction of amyloid, improve focus and alertness, improve the levels of NGF, stimulate neuronal branching, aid in detoxification, and boost the immune system are recommended for the prevention or treatment of AD [246,248,249,256,257].

Supplements are also a very important contributor to healing for those suffering from specific deficiencies that affect cognitive health. Long-term supplementation with anti-oxidant vitamins and mineral supplements is the most promising area for future research. Supplements including but not limited to ß-carotene, vitamin B12, folate (vitamin B9), vitamin B6, vitamin C, vitamin E, selenium, zinc, omega 3-fatty acids, glutathione, coenzyme Q10, alpha lipoic acid, choline, phosphatidylserine, and acetyl-L-carnitine may (a) improve short term memory in aging patients who have difficulty with recall, (b) improve the memory of patients with SCI or MCI, (c) help reverse some of the degenerative changes in brain function, and (d) prevent age-related mental decline and slow the progression of AD [258,259,260,261,262,263,264,265,266,267,268,269,270].

Thus, as one part of a comprehensive protocol, high-quality herbs and supplements tailored to the specific, evolving needs of each individual with SCI, MCI, or AD have proven to be important in treating or reversing AD [2,18,19,22].

## 3. Conclusions

Alzheimer’s disease is now the fifth leading cause of death for adults aged 65 and older and the most common cause of dementia among older adults [1]. Research studies have indicated one of the major risk factors for late-onset AD is gender, with postmenopausal women contributing to over 60% of all those affected. Based on the fact that women comprise approximately two-thirds of all AD patients, researchers have put forward the “estrogen hypothesis,” which explains how 17β-estradiol exerts a neuroprotective effect by protecting the female’s brain from AD development. This hypothesis is supported by recent findings showing estradiol’s role in signaling and transcriptional pathways involving cognition and memory. While more work is needed to understand the mechanism of estradiol’s neuroprotective action in AD, recent data lend support to the use of hormone replacement therapy (HRT) as a successful intervention for women at risk for AD [271,272,273,274].

Furthermore, ethnicity also has a role in AD, with African Americans and Hispanics at greater risk than whites to have AD. While African Americans are about two times more likely than whites to have AD, Hispanics are about one and one-half times more likely than whites to have AD and other dementias. While more studies are needed to understand the mechanisms responsible for these differences, better management of these risk factors may help reduce the risk of AD among women, African Americans, and Hispanics [275,276,277].

While the development of effective AD treatment and prevention is a major healthcare goal for all people at risk for AD, thus far, billions have been spent on research and clinical trials, and there is still no mono-therapeutic drug(s) to delay or reverse AD [15,16,278]. The recently approved FDA drugs have failed to show any significant slowing down of the actual symptoms of AD [279]. The drugs for Alzheimer’s that have failed are based on the concept that removing the amyloid would ameliorate AD symptoms. The various amyloid-removing drugs may have reduced the amyloid levels but failed to improve cognition. There may be several reasons for such repeated failure: (1) Treatment for AD is typically initiated late in the pathophysiological process; (2) Alzheimer’s disease is not a single disease but rather exhibits several different subtypes; and (3) AD is a complex chronic condition, and there are several potential contributors to AD, such as inflammation, various chronic pathogens, trophic withdrawal, insulin resistance, vascular defects, trauma, and exposure to specific toxins [2,15,16,18,19,22].

Table 1 illustrates the criteria for a perfect treatment for AD and suggests that a mono-therapeutic approach is likely to be suboptimal; instead, a personalized, multifactorial program based on each individual’s genetics and biochemistry may be preferable, as shown in Figure 1 and Table 2 [2,18,19,22]. Research studies from several independent groups have now revealed an extensive network of molecular interactions involved in AD pathogenesis, suggesting that a network-based therapeutic approach that addresses all the potential contributors to cognitive decline simultaneously, rather than a single target-based approach, may be more effective for the treatment of dementia or MCI due to AD [2,19,23,280,281].

In all these cases, the root cause(s) of the degenerative process is/are being targeted. Thus the AD pathogenesis itself is impacted, resulting in a sustained improvement that represents a major advantage over mono-therapeutics [5,18,21,22,23,24,25,26,27].

The management strategies to treat/reverse cognitive decline include but are not limited to diet, physical exercise, sleep, stress management, brain exercise, detoxification, herbs, and supplements, as shown in Table 1 and Table 2 and Figure 1. A balanced dietary approach utilizes multiple mechanisms to support the brain optimally to prevent and reverse cognitive decline by mechanisms such as increased energy, insulin sensitivity, reduced inflammation, improved vascular health, and detoxification [2,19,32,33,34,35,36,37,38,39,40]. Physical exercise is one of the best ways to reverse cognitive decline. It improves oxygenation, insulin sensitivity, and sleep; reduces overall stress; optimizes BMI (body mass index); and improves overall brain and body physiology [60,63,64]. Obstructive sleep apnea and other causes of poor oxygenation are risk factors for poor cognitive health. Sleep is vital for memory consolidation and promotes metabolic health, reduces inflammation, and upregulates the immune system [95,96,97]. In addition, people who engage in cognitively stimulating activities have a lower risk of cognitive decline and dementia. Mental stimulating tasks improve thinking, problem-solving, reasoning, and memory. Improvement is also seen in activities of daily living [122,142,144]. Stress, especially chronic, unresolved, or severe stress, is another key contributor to AD [160,161,162]. Stress management practices are required to reverse stress-associated cognitive decline [83,164,169,170,171,172]. The detoxification of toxins and avoiding exposure to bacteria, viruses, or mold, which are turning out to be major contributors to cognitive decline, especially in genetically susceptible individuals, is a vital part of optimizing brain health [193,194,195,196,197]. Herbs and supplements are needed to address any specific nutritional deficiency that affects cognitive health and, thus, can be a very important contributor to reversing cognitive decline [248,249].

Thus, results to date suggest that to successfully treat SCI, MCI, or AD, a mono-therapeutic drug strategy may not be optimal; instead, the most pragmatic approach involves addressing the above-mentioned targets underlying AD pathophysiology simultaneously. In other words, a network-based, multi-therapeutic approach may be feasible and potentially more effective (Table 2). While each of these strategies has been shown to reverse cognitive decline and promote neuroplasticity, when practiced together, their combined effect may be additive or even synergistic, and the benefits may be sustained, leading to overall health optimization and improved cognition.

## Figures and Tables

**Table 1:** Criteria for a perfect AD drug. A perfect drug is one that increases and optimizes all parameters on the left as they tend to be lowered or down-regulated in AD. The same drug decreases, lowers, or reduces the parameters on the right side, as these tend to be elevated in AD. The highlighted items indicate the targets of preference for most pharmaceutical companies. While some drugs are designed to target amyloid, others are focused on tau. However, here again, it is a mono-therapeutic strategy, and past single target-based failures have cast doubts on this approach.

Increase/Optimize	Decrease/Prevent/Optimize
APP α-cleavage	homocysteine
Neprilysin	APP β-cleavage
IDE	APP γ-cleavage
Aβ clearance	Caspase-6 cleavage
Autophagy	Caspase-3 cleavage
BDNF	APP b-oligomerization
NGF	P-tau and PHF
Netrin-1	Oxidative damage and ROS production
ADNP	NFkB
SIRT1	Glial scarring
PP2A activity	Inflammation
Phagocytosis	Synaptoclastic signaling
Insulin sensitivity	Neuronal cell death
Axoplasmic transport	
Mitochondrial function	
Cholinergic neurotransmission	
Long-term potentiation	
Vit D, B12, and Zinc	
Resolvins	
Detoxification	
Vascularization	
cAMP	
Glutathione	
Estradiol, progesterone, pregnenolone, DHEA, GABA, free T3, free T4, TSH	

**Table 2:** Reversing AD-associated cognitive health requires addressing potential contributors to cognitive decline with a personalized, multi-therapeutic approach. Each of the strategies mentioned in the table has the ability to improve cognition and brain health, and when practiced together, they create a powerful synergy with sustained improvement.

Multi-Therapeutic Strategies	Goals
**Nutrition**	Improves cognition and supports brain health by creating insulin sensitivity promoting metabolic flexibility/ ketosis reducing inflammation improving vascular health promoting autophagy
	
**Exercise**	increases brain-derived neurotrophic factor (BDNF) that stimulates neuroplasticity increases cerebral blood flow and oxygenation mitigates overall stress optimizes body mass index (BMI) improves insulin sensitivity reduces inflammation stabilizes brain volume and decreases white matter lesions
	
**Sleep**	enhances ability to focus, learn, and memorize reduces stress promotes neuroplasticity improves waste-clearing capacity
	
**Stress Management**	activates parasympathetic arm of HPA axis and balances stress hormones increases cerebral blood flow and oxygenation improves insulin sensitivity reduces inflammation boosts cognitive acuity
	
**Mental exercise** **(Brain stimulation)**	promotes neural connectivity in response to new learning improves mood, thinking, hearing, problem-solving, reasoning, and memory lowers risk of cognitive decline and dementia improves activities of daily living
	
**Detoxification**	Improves cognition and supports brain health by treating dementogen exposure and limiting ongoing exposure reducing systemic and brain inflammation optimizing gut, oral and nasal microbiome upregulating the immune system and mitochondrial energetics
	
**Herbs & Supplements**	Improve cognition and support brain health by promoting neural connectivity and effective synaptic support optimizing trophic support upregulating the immune system and gut health reducing inflammation boosting cognitive acuity improving vascular health neutralizing free radicals
	

## Data Availability

Not Applicable.
